# Integrating multi-scale data on homologous recombination into a new recognition mechanism based on simulations of the RecA-ssDNA/dsDNA structure

**DOI:** 10.1093/nar/gkv883

**Published:** 2015-09-17

**Authors:** Darren Yang, Benjamin Boyer, Chantal Prévost, Claudia Danilowicz, Mara Prentiss

**Affiliations:** 1 School of Engineering and Applied Sciences, Harvard University, Cambridge, MA 02138, USA; 2 Laboratoire de Biochimie Théorique, CNRS UPR 9080, Univ Paris Diderot, Sorbonne Paris Cité, IBPC, Paris, France; 3 Department of Physics, Harvard University, Cambridge, MA 02138, USA

## Abstract

RecA protein is the prototypical recombinase. Members of the recombinase family can accurately repair double strand breaks in DNA. They also provide crucial links between pairs of sister chromatids in eukaryotic meiosis. A very broad outline of how these proteins align homologous sequences and promote DNA strand exchange has long been known, as are the crystal structures of the RecA-DNA pre- and postsynaptic complexes; however, little is known about the homology searching conformations and the details of how DNA in bacterial genomes is rapidly searched until homologous alignment is achieved. By integrating a physical model of recognition to new modeling work based on docking exploration and molecular dynamics simulation, we present a detailed structure/function model of homology recognition that reconciles extremely quick searching with the efficient and stringent formation of stable strand exchange products and which is consistent with a vast body of previously unexplained experimental results.

## INTRODUCTION

**Figure 1.**
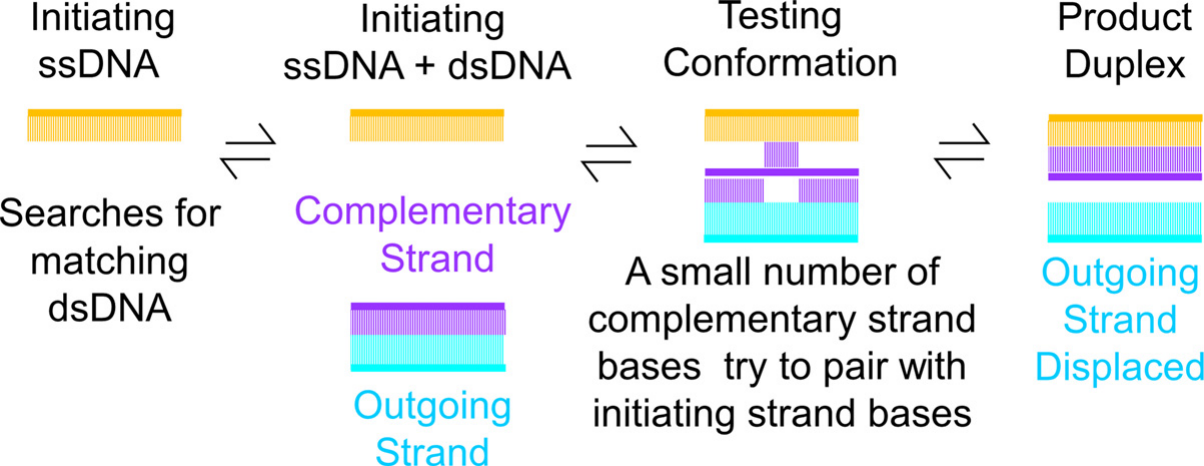
Schematic of the homology searching process. The initiating, complementary, and outgoing strands are shown in orange, purple and cyan, respectively. RecA protein monomers are excluded for simplicity.

Homologous genetic recombination (HR) is a prescribed and necessary part of the DNA metabolism of every free-living organism. Recombination can accurately repair DNA double strand breaks, provides crucial links between pairs of sister chromatids in eukaryotic meiosis, and contributes in smaller ways to a host of additional cellular requirements. All of this is centered on the function of RecA-class recombinases and their capacity to catalyze (i) an alignment of homologous sequences in one single-stranded DNA (ssDNA) and another double-stranded DNA (dsDNA) and (ii) the transfer of one strand of DNA from the duplex to the initiating ssDNA leading to the formation of a stable heteroduplex if the ssDNA and dsDNA are homologous. The latter process is referred to as strand exchange and is illustrated schematically in Figure 1.

**Figure 2.**
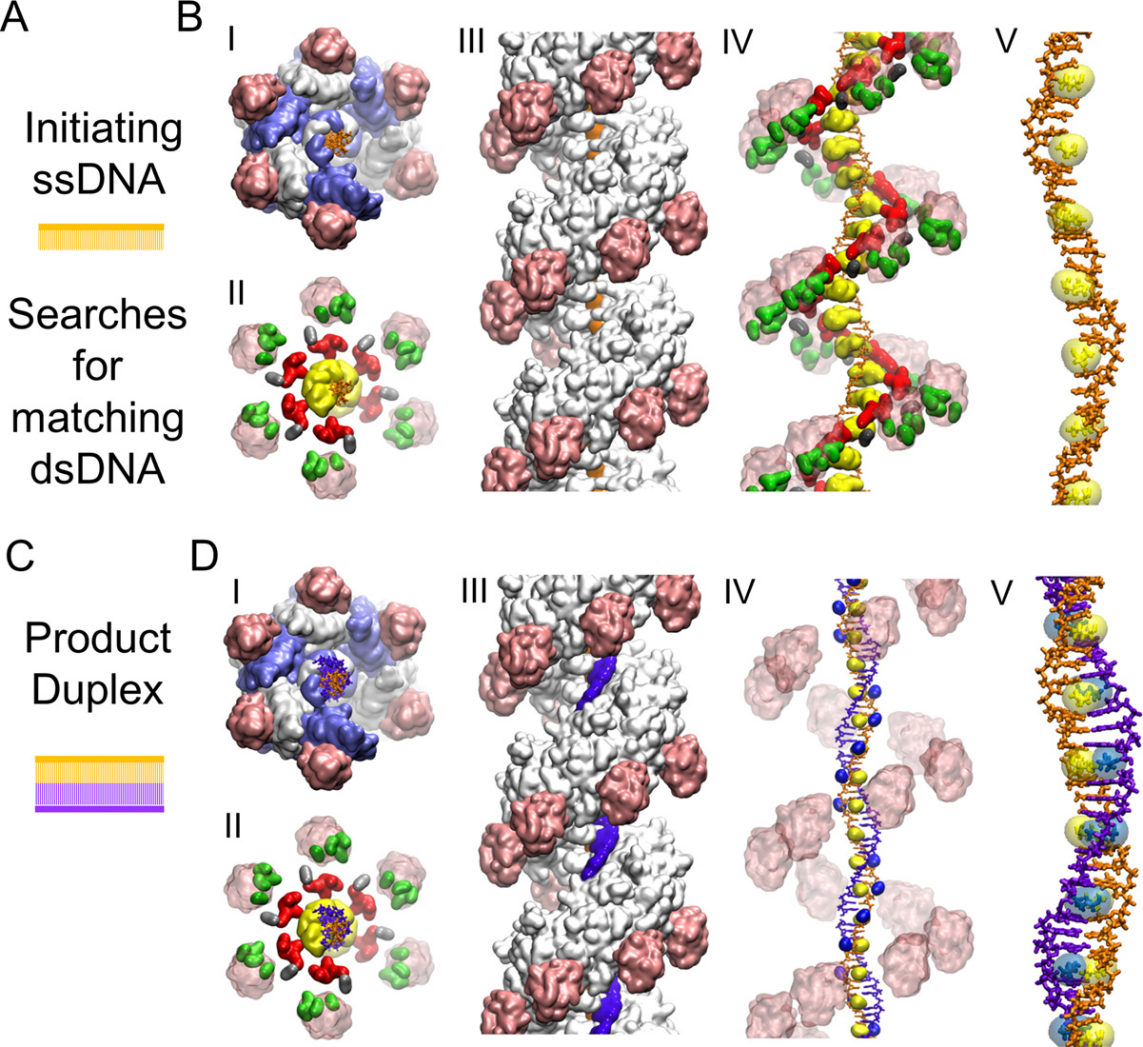
Structures known from crystallography. The initiating and complementary strands are shown in orange and purple, respectively. The residues in the C-terminal domain are shown in pink. (**A**) Schematic showing the initiating ssDNA. (**B**) I. Active filament seen from the bottom with the protein with alternating monomers shown in white and blue. (**B**) II. The residues in the C-terminal domain are shown in transparent pink except for lysine residues K280, K282, K286 and K302, which are shown in green. K232 is shown in silver. Site II residues R226, R227, R243, and K245 are shown in red, and the L2 loop (198–206) is shown in yellow. (**B**) III. Same as I., but seen from the side with the 5′ end of the initiating strand at the top. (**B**) IV. Same as II., but from the same angle as (B-III). (**B**) V. Close-up of the initiating strand and I199. (**C** and **D** (I. to III)). Analogous to (**A**) and (**B**), respectively. (**D**) IV. shows I199 in yellow and M164 in royal blue highlighting their intercalation in the rises in the initiating and complementary strands, respectively. (**D**) V. is analogous to V. in (**B**), showing M164 (royal blue) intercalating in the rise in the complementary strand.

RecA protein, found in essentially all bacteria, is the prototypical recombinase. Homologs exist in other organisms, including RadA in archaea and Rad51 and Dmc1 in eukaryotes. An outline of the mechanism by which these proteins align homologous sequences and promote DNA strand exchange is in hand (1–3), and RecA will be used to illustrate it. First RecA protein forms a presynaptic active filament by polymerizing onto an initiating ssDNA. The resulting filament is a right-handed helix, with six RecA subunits per turn and three nucleotides of DNA bound per subunit with the initiating ssDNA bound deep within the helical groove of the filament, in a location referred to as the primary DNA-binding site (site I) (Figure 2A and B). The filament then interrogates any nearby dsDNA to find a homologous match. During the search, the dsDNA enters the helical filament groove and is initially (usually transiently) tethered. Once sequences are aligned, the sampling required to sense homology involves transient destabilization of a short region within the dsDNA and limited base-flipping within that short region (4,5). If the flipped bases in the complementary strand match the corresponding bases in the initiating ssDNA, the pairing of the complementary strand substrate is transferred to the initiating ssDNA resulting in the formation of the product duplex, while the complementary strand's original pairing partner, the outgoing strand, is displaced (Figure 1). The beginning and endpoints of this process, the bound initiating ssDNA, and the product dsDNA bound to site I have been structurally defined through crystallography (6) (Figure 2C and D). In both cases, the bound DNA strands are extended to about 1.5 times the B-form dsDNA length and present a very specific conformation, where the extension is not uniformly distributed; instead, the bases are grouped into stacked nearly B-form groups of three consecutive base pairs (triplets) that are separated by large rises. The B-form triplets are compatible with the overall extension and untwisting because the extension and untwisting occur dominantly in the rises between the triplets.

Experimental work suggests that strand exchange is divided into stages characterized by very different strange exchange rates. In particular, single molecule measurements of the strand exchange rate indicating that strand exchange slows drastically after a very rapid initial interaction (7). The very rapid early stages of homology recognition are very difficult to capture experimentally (5,7); however, both bulk (8) and single molecule experiments (9,10) have captured strand exchange after the initial rapid interaction. Those experiments indicate that after the initial rapid interaction, strand exchange progresses at a characteristic rate that is insensitive to dsDNA length and ATP hydrolysis, but dependent on temperature. At 37°C the characteristic rate is ∼ 6 bp/s, whereas at 22°C it is ∼2 bp/s (8–10).

The division of homology recognition into stages where the first stage tests ∼8 bp is consistent with measurements suggesting that the strand exchange product remains highly unstable until the length of the product extends to 8–9 consecutive base pairs, after which there is a sudden increase in the stability of the product in both RecA (11) and Rad51 (12). This greatly increased stabilization may result from a transition to a metastable conformation whose strand exchange rate is much slower than the strand exchange rate for the initial interaction. Both RecA (11) and Rad51 (12) experiments have shown that after the initial rapid interaction each strand exchanged triplet increases the stability of the strand exchange product until an asymptotic binding value is approached when 15–20 contiguous homologous bp occupy the strand exchange conformation. The homology recognition and strand exchange process that leads to the formation of 20 bp strand exchange products can occur without ATP hydrolysis (13); however, the unbinding of the heteroduplex product dsDNA from the protein requires ATP hydrolysis (9).

Seminal early work suggested that RecA might use an initial very stringent homology test of ∼8 bp, that is followed by a less stringent testing stage (14). Recent experimental work indicates that an initial ∼8 bp test that accepts ∼1 mismatch in eight bases is followed by a less stringent testing stage that allows homology recognition to pass over non-homologous triplets within otherwise homologous sequences (15). The progression of strand exchange through mismatched triplets in otherwise homologous sequences was also observed in previous work (16).

Experimental (17) and theoretical studies (18) suggested that dsDNA tension could provide a physical mechanism that divides the search into stages since the tension creates an unfavorable binding energy contribution that increases non-linearly as a function of the number of base pairs bound in the positively charged helical track within the filament groove (Figure 2B-II and B-IV) known as the secondary DNA-binding site (site II) (18). That previous work (18) proposed that favorable interactions between the dsDNA and the presynaptic filament drives rapid binding of 2–3 base pair triplets to site II. The binding of two or more triplets to site II extends the outgoing strand more than the complementary strand, creating a mechanical stress on the base pairs joining the complementary strand to the outgoing strand backbone. That stress destabilizes the base pairing in the dsDNA, leading to rapid base flipping and homology testing of ∼8 bp. In this model, dsDNA tension prevents more bases from binding to site II unless the ∼8 bp that are tested initially make a homology dependent transition to a metastable conformation. If the initial test is passed, the system makes a transition to a metastable conformation (18).

Recent theoretical work (19,20) suggests that a two stage system could overcome the general speed stability paradox that arises because stringency requires correct interactions to be strongly bound while speed requires that nearly correct interactions be easily reversed. More importantly, the theoretical work suggests that the two stage system could search more than 10^5^ bp in less than 1000 s, while non-homologous pairings efficiently form stable strand exchange products (20,21). In the system, the searching speed is provided by the initial rapid test that rejects ∼95% of all pairings before they progress to the slower testing stage. Only rare pairings that pass that initial test would progress to the second slower testing stage that is characterized by the slow strand exchange rate (20,21). This is consistent with recent experimental studies showing that 20-nt sequences presenting less than 15 matches (which represent the large majority of accidental matches in vivo) do not lead to the formation of any detectable metastable intermediate during strand exchange, while rarer accidental sequence matches lead to the formation of metastable strand exchange products before being rejected (15).

The early stage of recognition during which strand exchange progresses rapidly encompasses much of the mystery of homologous recombination: what structural features allow the search to form the correct homologous pairing in much less than an hour? In principle, molecular modeling should be an appropriate method to fill the structural gap between the beginning and endpoints of the recognition and strand exchange process, while integrating the large experimental information that has been accumulated along the last decades. However, modeling such a large and complex system remains a formidable challenge for current computational capacities. Early studies considered only nucleic acids without including interactions with the protein. Those studies successfully explored how changes in DNA properties due to RecA-induced stretching distortion may contribute to the mechanism (22–24). However, the whole nucleofilament system must be considered in order to understand which structural features in the DNA-filament association drive strand exchange forward for homologous sequences while rapidly halting the process in case heterologous sequences are encountered.

Recently, Saladin *et al*. (25) have investigated possible association geometries at early stages of the recombination process using a multi-scale strategy consisting of coarse-grained extensive docking search, interactive simulations of selected docking geometries, and atomic molecular dynamics simulations. This work showed that combined state-of-the-art modeling methods can now tackle very complex systems that are out of reach of classical modeling methods (26). Due to the high dimensionality of the systems under study, such strategies gain in efficiency if the search space can be restricted using available experimental information such as low resolution data or information obtained at the residue level. In the case of polymeric systems such as the active filament, the multiplicity of identical residues in similar environments within each monomer makes it difficult to restrict the search space based on the proximity to given residues: one particular interacting residue with functional role cannot be easily distinguished from the non-interacting instances of that same residue. If in addition the system is dynamic like in recombination, it is generally not known in which step of the reaction a given residue is involved. Models of the recombination system must therefore be guided by other considerations than the residue information, which can nevertheless be used for a posteriori validation.

In their study, Saladin *et al*. resolved to look for direct interactions between the bases of the searched dsDNA and the initiating ssDNA as early contact points between the dsDNA and the filament. In this scheme, the strength of dsDNA binding to the filament would directly depend on sequence homology. The study concluded that such mode of direct interaction involving short segments of three consecutive bp is possible provided that the DNA structure adopts a conformation curved towards the major groove upon binding the filament and that at least one long RecA loop (L2 loop, residue 198 to 206, shown as yellow in Figure 2B-II and B-IV) is displaced (25). It is compatible with a propagation of the reaction where groups of three dsDNA base pairs would successively unroll and bind the corresponding ssDNA bases via homology-dependent Watson-Crick interactions, while displacing one L2 loop at a time. Stabilization of such system would increase linearly when more homologous triplets bind and decrease if at least one bp does not match.

This model has many desirable features; however, the timescale suggested by the model is slow since at least one L2 loop needs to be displaced at each search event involving three bp, so it is unlikely that millions of search events take place in less than an hour. However, in the context of a two-stage process discussed above, the 3 bp-progression characterizing the Saladin model may be compatible with the second (slow) phase of strand exchange progression, where mismatches that have passed the rapid initial testing are eliminated.

In the present work, we explore the geometries that may characterize the first rapid stage by investigating an alternative route to sequence probing and strand exchange. Given the steric configuration of the filament groove (6) the route via site II is in fact the only alternative to the direct base contact previously explored. The modeling followed the outlines of the Saladin et al. former work with, however, some noticeable additional challenges since no structure at the atomic level is available for the stretched and unwound distorted DNA structure induced by its binding to site II (5) (this structure necessarily differs from the endpoint DNA structure resulting from strand exchange).

The initial search for geometries of dsDNA association to the filament therefore needed to simultaneously search for favorable docking geometries and generate DNA structural distortions that would optimize the association. Yet, the few published flexible docking methods that account for DNA flexibility during the docking search either limit the DNA deformations to curvature (27) or explicitly rely on experimental information at the residue level to restrict the search space (28). Since neither approach could be used here, we developed a multi-step strategy for this part of the modeling, which can be related to the approach by Banitt and Wolfson as it includes the docking of DNA fragments, but which takes advantage of the helical periodicity of the system and allows any type of distortion. More generally, the search was guided by principles issued from recent theoretical studies of recognition at the genomic scale (20,21) as well as previous modeling work on distorted DNA (23,29–35). Finally, when submitted to all-atom molecular dynamics simulations in solvated environment, the resulting model spontaneously gave way to pairing exchange. Importantly, without any guiding, the structure assumed by the strand exchange bases corresponds to the structure known from crystallography. This spontaneous evolution towards the known structure of the strand exchange product provides substantial support for the validity of the modeling that precedes it.

We will end by discussing functional implications of structural features revealed in this work, some of which we mention briefly here. Importantly, the model suggests that the tension resulting from the extension of the dsDNA bound to site II naturally divides the search into stages, where the first stage rapidly tests ∼ 8 bp. Furthermore, the unanticipated iterative flipping of successive base pair duplets provides previously unexpected advantages in searching speed. Finally, the work suggests a possible origin for the large increase in stability that is observed after 8 contiguous bp have undergone strand exchange.

## MATERIALS AND METHODS

### Overall strategy

The construction of intermediate states of DNA association to the nucleofilament active for recombination followed a combination of one or several modeling steps described below:

- Scanning the possible modes of DNA/filament association via docking simulations; when the association was expected to induce distortions in the DNA structure, the scanning step was refined either by using different (curved) forms of the DNA or by docking small DNA fragments, followed by reconstruction into a complete DNA;
- Constructing DNA structures either with helical symmetry or where different DNA regions interact in different ways with distinct regions of the RecA filament, using the PTools/Heligeom computing tools and restrained energy minimization;
- Refining the modeling results using short all atom, fully flexible molecular dynamics (MD) simulations;
- Simulating the dynamic evolution of the refined model during longer MD trajectories.

### Docking simulations

Rigid body docking of B-form DNA to the filament (receptor) was performed using either Autodock Vina (36) or the ATTRACT program (29,37) in its PTools/PyAttract implementation (25,38). These simulations explored the possible mode of association of oligonucleotides to the filament as a gateway to filament incorporation. In both cases, the B-DNA structure (ligand) was docked on a protein nucleofilament composed of five RecA monomers bound to ssDNA (PDB code 3CMW) (6) (receptor). Autodock Vina considers the association partners in atomic representation and uses docking scoring function developed based on X-Score (39). For this simulation, the whole filament surface was selected as putative binding pocket on the receptor surface. The ATTRACT docking simulations in coarse-grained representation followed the same protocol as detailed in reference (25); however, the simulations done here include the L2 loops that had been omitted in that previous work. The two simulations converged towards DNA binding in the filament groove as described in the Results section.

Flexible docking approaches were used to model the DNA incorporated in the filament since the stretching/unwinding distortions induced by its binding to the filament were not structurally elucidated at the atomic level. The docked DNA was either a single-stranded or a double-stranded DNA with sequence homologous to that of the RecA-bound single strand in the crystal structure (i.e. (dT.dA)_n_). Three different protocols were used to explore the possible interaction sites, and two protocols were used for subsequent construction. Using different protocols aimed at avoiding any possible dependency on the flexible docking method, given that the docked structure presents uncommon distortions and length-dependent internal stress.

Two out of the three docking protocols started by a rigid body docking run as detailed above. In the ATTRACT protocol, the docked ligand was a curved (dT.dA)_30_ DNA built from the structure of SRY-bound DNA (PDB code 1HRY) (described in (40)), and the simulations targeted the whole filament surface, again in the presence of the L2 loops. The best interacting patches on the protein surface were situated in a cleft between the L2 loop and the filament interior, containing the positively charged protein residues K198, K216, R222, R226, R227, R243 and K245 associated to site II in the literature (6). In the Autodock Vina protocol, the docked ligand was a short DNA fragment composed of a 3-base-pair (bp) long nucleotide phosphate-deoxyribose, and the simulations targeted the putative site II basic patches described above. A third protocol explored the positioning of a preliminary stretched-unwound single-stranded DNA in the site II cleft using interactive simulations with the BioSpring simulation motor described in (25), with the help of a haptic device. In this case, both the filament and the DNA were considered in coarse grained representation, and internal flexibility of the DNA and selected protein regions (L2 loops and residues lining site II, i.e. R143, R145, K226, K227) were accounted for using an augmented spring network (25). Each of the three protocols resulted in identifying the best interacting regions on the protein surface and the best interacting trinucleotides along the DNA structure; the local geometries associated to the most favorable interaction energies were used for further modeling.

Modeling the DNA bound to the identified sites followed two main protocols. The first one consisted in reconstructing full single- or doubled-stranded DNA structures starting from the protein-bound trinucleotides selected in the previous step, taking into account the stoichiometry of association of three nucleotides per RecA monomer. More precisely, these geometries were replicated at each equivalent region along the filament, following its helical symmetry, and the DNA trinucleotide single- or double-stranded fragments were linked together using energy minimization of the phosphodiester backbone geometry under harmonic restraints on the interacting phosphate atoms to maintain the interactions with their protein residue partners. This was followed by short MD simulations (see below) and annealing of a second strand when the starting DNA fragment was single-stranded. Alternatively, a whole (dT.dA)_9_ DNA oligomer obtained by truncating the site I-RecA-bound dsDNA structure in the 3CMX, already presenting 50% stretching and 40% unwinding deformations with respect to B-DNA, was fitted to the site II patches identified in the first step. To this aim, harmonic restraints were applied to the phosphate atoms of thymine residues 1, 2, 4, 5, 7, 8 during energy minimization with the Jumna software (29) to radially pull these phosphate atoms to the favorable positions identified in site II under constant helical symmetry conditions (i.e. conserving the global stretching and winding characteristic of the binding to the site I). This process was applied to DNA in the absence of the protein to capture the contribution of the DNA internal mechanics to the protein-DNA association geometry.

### Molecular dynamics simulations

Molecular dynamics (MD) simulations were performed using the NAMD 2.9 package (41) with the CHARMM 27 force field including CMAP correction (42). Solvation of the simulated structures used TIP3P water model placed within a 125 Å × 120 Å × 170 Å box, subject to periodic boundary condition. Physiological concentration of Na^+^ and Cl^−^ ions of 0.15 mol/l were added to each simulation to maintain electro-neutrality (Supplementary Figure S1A). As a whole, the simulated systems contained over 260 000 atoms. We used 2-fs time steps, and the bond lengths involving hydrogen atoms were constrained using the SHAKE method. We utilized particle-mesh Ewald method for long-range electrostatics calculation, and van der Waals interactions were smoothly switched off at 10–12 Å by a force-switching function. The temperature and pressure were maintained along the simulation using a Langevin dynamics scheme and a Nosé–Hoover–Langevin piston, respectively. We performed MD simulations on several RecA/ssDNA/dsDNA complexes resulting from the docking and construction steps (see above). These structures were first energy minimized using 50,000 conjugate gradient energy minimization steps followed by slow heating from 30 to 300 K over 500 ps. During these preparation steps, the α-carbon and phosphate atoms of the protein and nucleotide were restrained using a soft harmonic potential with a spring constant of 0.5 kcal mol^−1^ A^−2^. The system was further allowed to equilibrate for 5 ns in the NPT ensemble with pressure of 1 bar and temperature of 300 K. During the equilibration process only the α-carbon atoms of the terminal RecA monomers and phosphate atoms of the three nucleic acid strands were restrained with a harmonic potential with slowly decreasing spring constant from 0.5 to 0.05 kcal mol^−1^ A^−2^. In the production simulations, the system was maintained under NPT ensemble conditions, and harmonic restraints of 0.05 kcal mol^−1^ A^−2^ only applied to the α-carbon of terminal RecA monomers and phosphate atoms of the terminal nucleotide of each nucleic acid strand. The simulation durations varied between 10 and 25 ns. In one case, an accelerated dynamics protocol was used after the first 10 ns of classical MD (43). For this phase of the simulation, a dual boost mode was used. The threshold energy E (kcal mol^−1^) and the acceleration factor α were respectively set to 21678, 1946 and −775729, 56772 for the dihedral potential and the total minus dihedral potential which were calculated as suggested by Markwick *et al*. (44). The root mean square deviation (RMSD) of the backbone and the energies during the heating, equilibration and production of the simulation are shown in Supplementary Figure S1B.

### Construction with PTools/Heligeom

Model construction was performed at different stages of the study; for example, to replicate DNA fragments during the flexible docking construction stage, when joining DNA stretches that interact in a different way with different regions of the filament or when issuing intermediate structures for the movie animation. Many of these DNA structure manipulations or helical transformations were performed using the PTools/Heligeom library (45).

### Movie

The movie was elaborated using PyMol scripts as described in the PyMol online tutorial (http://www.pymolwiki.org/index.php/MovieSchool). Scene making combined PyMol built-in facilities such as interpolation between different views (for initial DNA approach, change of viewing angles or zooming) and the construction of intermediate structures (first part of the movie) or structures taken from a smoothed MD trajectory (second part).

## RESULTS

In this work, we modeled intermediate species along the early process that goes from non-specific DNA uptake by the filament to the very first pairing exchange events. These events entirely take place during the first, very rapid stage of HR. We divide the overall DNA/RecA structural changes along that early process into four major conformational classes: (i) presynaptic active filament, (ii) bound B-form dsDNA, (iii) conformations with dsDNA distortions stabilized by interactions with site II, and (iv) postsynaptic filament with the complementary strand paired with the initiating strand bound to site I.

The structure of the presynaptic active filament (class 1), which consists of the RecA subunit polymerized onto the initiating ssDNA, was determined through crystallography (6) (Figure 2B and D). The initiating ssDNA is bound deeply in site I in the region where the L1 and L2 loops meet. Its structure, along with that of the protein filament, is almost identical in the presynaptic and the postsynaptic filaments (6), as Figure 2 illustrates.

### Non-specific binding of B-form dsDNA to the RecA filament

The structure of the non-specific binding intermediate with unaltered DNA structure (class 2) was modeled as the result of rigid body docking of B-DNA to about one turn of the presynaptic active filament using either the Autodock Vina in all-atom representation or the ATTRACT program in coarse-grained representation (see ‘Materials and Methods’ section). The two protocols converged towards structures where the DNA is partially inserted in the filament groove and where at least four DNA phosphates strongly interact with residues K280, K282, K286, and K302 through the formation of salt bridges. In these structures, which remained stably assembled during 20 ns of MD simulations (Supplementary Figure S2), the interacting phosphates are distributed in the two DNA strands while the basic protein residues belong to two consecutive or near-consecutive C-terminal domains (CTD) of RecA. Two examples of such structure are shown in Supplementary Figure S3.

### Non-specific dsDNA binding to the filament site II

The presence of bulky L2 loops folded upon the ssDNA in site I of the RecA filament (residues 198–206 shown in yellow in Figure 2B) only offers two possibilities for the dsDNA alignment in parallel with the ssDNA, either on the ssDNA side of the L2 loops or on the opposite side (Supplementary Figure S4). The first possibility has been explored in former theoretical work (25) which concluded that at least one L2 loop needs to be displaced for each dsDNA triplet to reach alignment with the incoming strand. In the present work, we explore the second route. The C-terminal side of the L2 loops forms a strongly electronegative cleft when intersecting with the protein core (Supplementary Figure S5), which can accommodate a DNA single strand as was confirmed here using interactive docking simulations with the BioSpring software (see ‘Materials and Methods’ section). This cleft, lined by clusters of basic protein residues R226, R227, R243 and K245, most of which are well-conserved among bacterial RecA proteins (46), was previously proposed to be the secondary DNA-binding site (site II) of the protein (6). In what follows, we will assimilate the cleft and its clusters of basic residues to site II. Indeed, the three flexible docking protocols with Autodock Vina, ATTRACT, or BioSpring (see Methods) that we used to model single-stranded or double-stranded DNA in site II in the presence of the L2 loops converged towards these clusters of basic residues. From the results of the docking simulations, we modeled the structure of DNA in site II as described in Methods.

**Figure 3.**
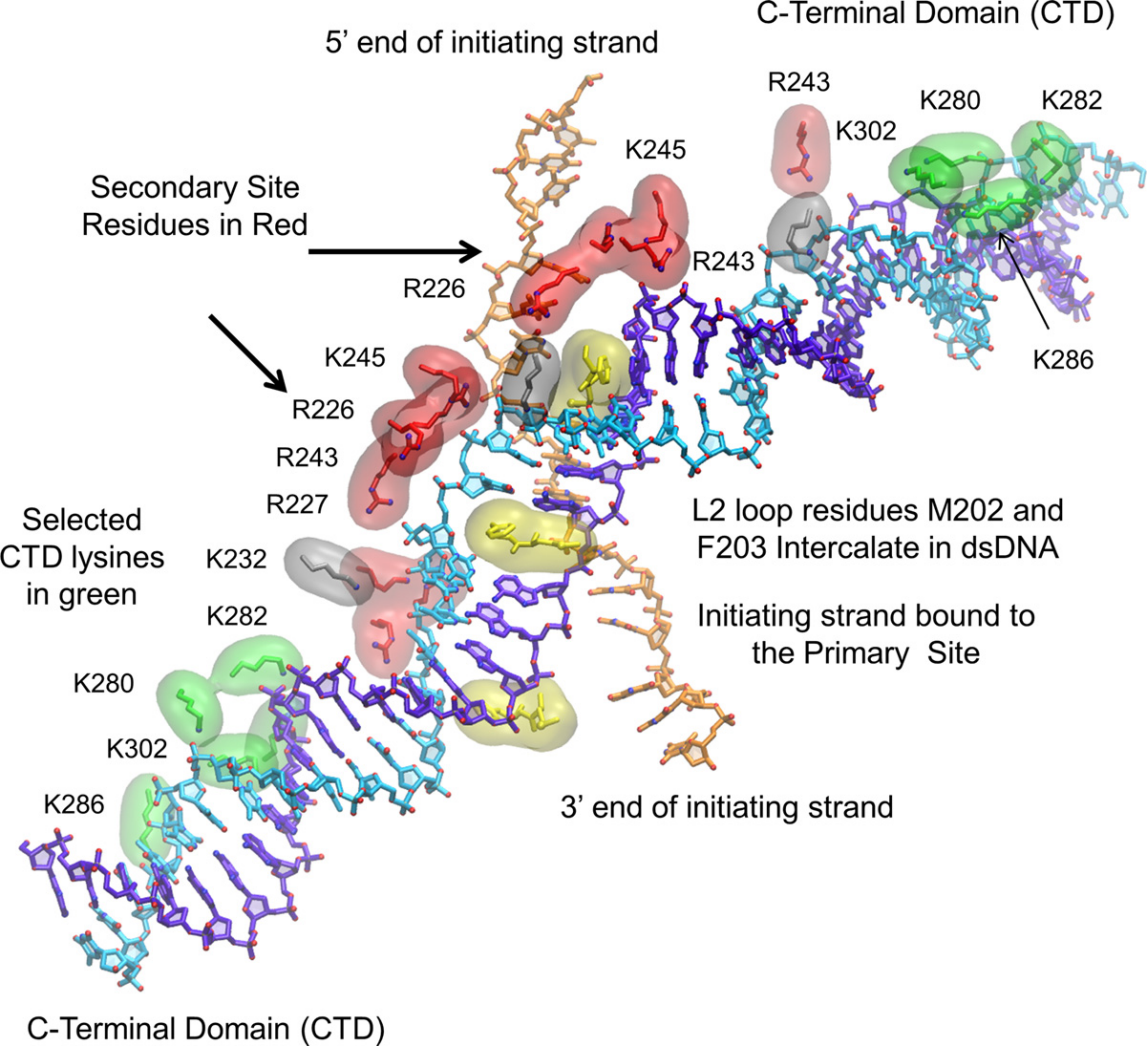
A detailed view of the active filament bound to dsDNA stabilized by interactions with RecA C-terminal domain and site II residues. The initiating, complementary, and outgoing strands are shown in orange, purple, and cyan, respectively. The lysine residues K280, K282, K286 and K302 are shown in green. K232 is shown in silver. The secondary DNA-binding site residues R226, R227, R243 and K245 are shown in red, and the L2 loop residues M202 and F203 are shown in yellow. The black arrows point to the site II residues at the beginning and end of the extended and untwisted region. F203 intercalates the bound dsDNA, and some simulations showed M202 can intercalate as well (see also Supplementary Figure S5).

In the resulting structure shown in Figure 3 (see also Supplementary Figure S5), each two consecutive phosphates from outgoing strand triplets strongly bind a cluster of basic residues R226, R227, R243 and K245 of each RecA monomer. Both the outgoing and the complementary strands directly contact many L2 loop residues including F203, and to a lesser extent M202, which notably intercalate in the rises between triplets supporting the extension.

The dsDNA in site II presents exactly the same helical characteristics as DNA bound to site I, with an average axially projected rise of ∼5.1 Å and an average twist of ∼20° per base pair step, consistent with the overall helical characteristics of the filament (Supplementary Figure S6). It ensures that the corresponding bases between initiating (in site I), complementary, and outgoing strands (in site II) can remain in registration, as suggested earlier (5). The DNA in site II is, however, much more stressed than the DNA in site I since the phosphodiester backbone of the outgoing strand is radially displaced from the helix axis by ∼15 Å, whereas the phosphate backbone of the initiating strand is only displaced by ∼9 Å (Supplementary Figure S6). Thus, the average distance between consecutive phosphate groups for the outgoing strand DNA bound to site II is ∼2 Å longer than the ∼5 Å separation for DNA in site I. Indeed, the phosphate extension of the outgoing strand DNA in site II approaches the maximum possible extension of the backbone (47). Such large extension prevents more than two of the three bases within a triplet from stacking as in B-form dsDNA, and, indeed, all MD simulations run on the system indicated constant evolution of the patterns of intra- or inter-strand base stacking, base-F203 stacking, and base pairing during the trajectories. The dynamic evolution of site II-bound dsDNA will be further described below.

### Junction between the dsDNA and the B-DNA fragment

The direction of the B-form dsDNA bound to the open filament groove substantially differs from that of the dsDNA bound to site II parallel to the helical axis. As a result, the junction between these two DNA regions can be expected to be bent. Similar expectation arises from structural considerations, as the junction between the stretched/unwound DNA distortions inside the filament and the B-form dsDNA at the filament entry is expected to produce a kinked conformation.

Indeed, previous work revealed a mechanical relationship between the type of global DNA distortion found in the RecA filament and the local distortion induced by DNA minor groove binding proteins such as SRY, TBP or HU (33,48). Applying pulling restraints to the 3′-extremities of the DNA region that contacts the protein either gave rise to the bending/untwisting distortions observed in case of single protein binding or to the stretching/untwisting distortions observed in the RecA filament (24,34). For this family of distorted DNA structures, kinks (and therefore stacking interruption) appear at junctions between DNA regions that shifted to the distorted state and the rest of the structure in B-form dsDNA. When the structural transition concerns the whole DNA structure like in the RecA case, the stacking interruptions turn into intercalation-like base pair separation.

Quite interestingly, recent investigation on the intercalation pathway of daunomycin to the DNA double helix identified a kinked DNA state as a metastable intermediate between a straight B-form state with daunomycin bound in the DNA minor groove and the intercalated state (35). In the case of dsDNA incorporation into the RecA filament, we propose that both types of stacking interruptions co-exist along the same DNA, either as a kink at the junction between the B-form dsDNA and the distorted regions, or as intercalation sites within the distorted region. This further suggests that a mechanism analogous to that of daunomycin intercalation may be a key component of the process of DNA extension in site II, as illustrated in Supplementary movie (scene 3). Experimental support for the presence of a sharp bend at the junction between the DNA outside the filament and the DNA inside the filament is provided by earlier experiments that imaged Rad51 mediated strand exchange and found that DNA incorporation into the filament during strand exchange is accompanied by a sudden change of direction, with measured angular values close to 90° (49).

Based on these observations, we modeled the junction region between the CTD-bound B-form dsDNA region and site II-bound stretched-unwound DNA region starting from the structure of DNA bound to the SRY protein (50). This DNA was first anchored to the filament interior using docking simulations as reported in Methods. The most favorable docking regions involved the bent region on the DNA side and a region largely overlapping the site II region and partially a CTD on the protein side. Specifically, the MD relaxed structure presented salt bridges between the basic protein residues R243, R245, K232, R226, R227 and DNA phosphates from both strands and on both sides of the curved region (Figure 3 and Supplementary Figure S5). Additionally, the aromatic ring of a phenylalanine residue F203 interacts in the DNA minor groove at the level of the kink, thus stabilizing the associated stacking interruption.

### Dynamic evolution of site II-bound dsDNA

We carried out extensive all atom MD simulations of dsDNA structures bound to one turn of the RecA-ssDNA filament in an explicit solvent environment (see Methods). Since the evolution of MD simulations depends on details of the starting structures, we performed several simulations with different starting structures, either restricted to the dsDNA region bound to site II or including the dsDNA B-form region bound to the CTDs and the junction region, with a number of bound triplets varying between one and five.

Some robust features of the homology search process emerge from the comparison of these MD simulations. First of all, all DNA strands remained bound to the protein during the course of the MD simulations extending over more than 20 ns, despite the large DNA distortions. Furthermore, if the structure contained B-form regions, the B-form dsDNA showed little structural evolution regardless of the number of triplets bound to site II (Supplementary Figure S2); however, whether or not B-form tails were present, the evolution of the region of the dsDNA that bound to site II showed an evolution that depended strongly on the number of base pairs bound to site II, where the dependence is consistent with a base pair stress that increases with the number of base pairs bound to site II. Supplementary Information includes a detailed discussion of the evolution of structures in which all of the dsDNA is bound to site II. That discussion concludes that such structures are not suitable for homology recognition and strand exchange.

As mentioned above, simulations of structures in which the site II bound base pairs are joined to B-form tails are consistent with base pair tension increasing with the number of site II bound triplets (Supplementary Figure S7). In particular, structures with only one triplet bound to site II rarely evolved. In contrast, when two triplets were bound to site II, one or two bases spontaneously flipped towards the minor groove, typically occurring within tens of nanoseconds after two consecutive triplets were bound in site II. The base flipping was followed by Watson–Crick pairing interactions between bases from the complementary strand and bases from the site I-bound ssDNA.

**Figure 4.**
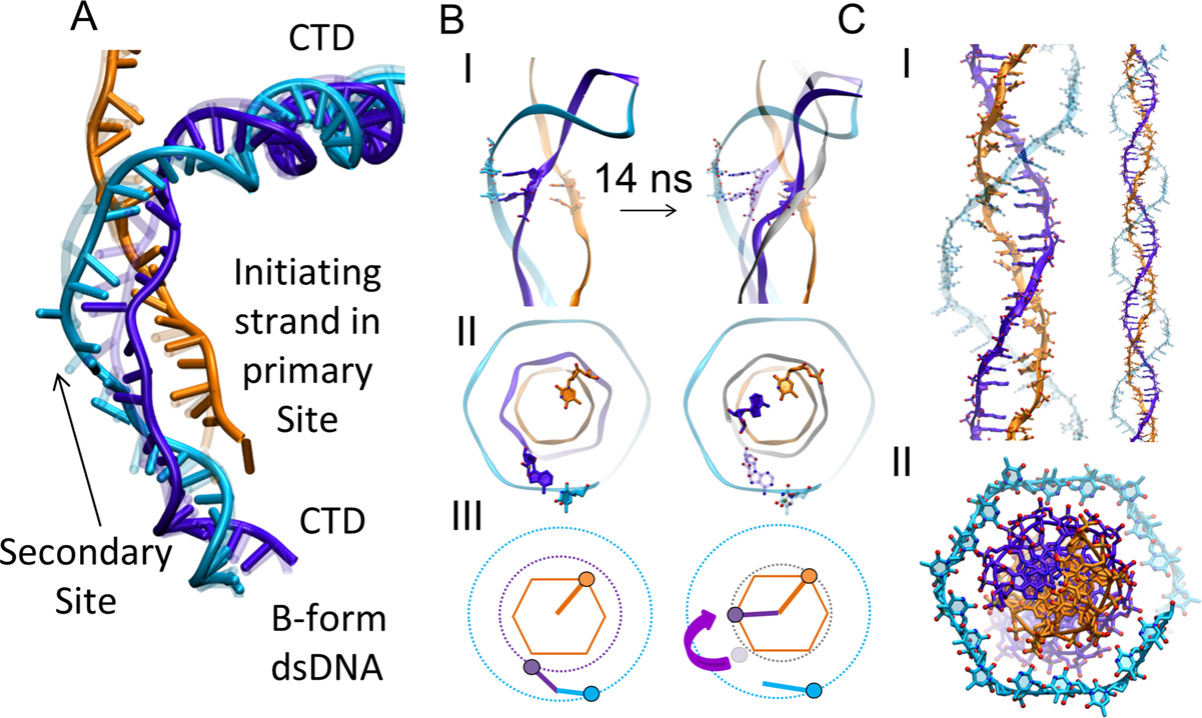
Illustration of base-pair homology testing using a duplet that is nearly in site I, while the third base of the triplet at the 3′ end of the triplet remains positioned in site II. (**A**) Structure of full filament with stretched and unwounded dsDNA bound to site II. The transparent and solid structures represent the conformations before and after 14 ns of simulation. (**B**) Structures before and after 14 ns simulation (10 ns conventional MD and 4 ns accelerated MD). (**B**) I. The complementary backbone in the postsynaptic filament is shown in silver. After flipping, the backbone near the flipped duplet shown in purple is nearly in postsynaptic position. (**B**) II. and III. Top and schematic views of the complementary and initiating bases at the 5′ end of a triplet before and after flipping. The transparent structures indicate the initial positions. The purple arrow indicates the relocation of the complementary strand backbone. (**C**) Postsynaptic dsDNA in site I; side view and top view are shown in I. and II., respectively.

**Figure 5.**
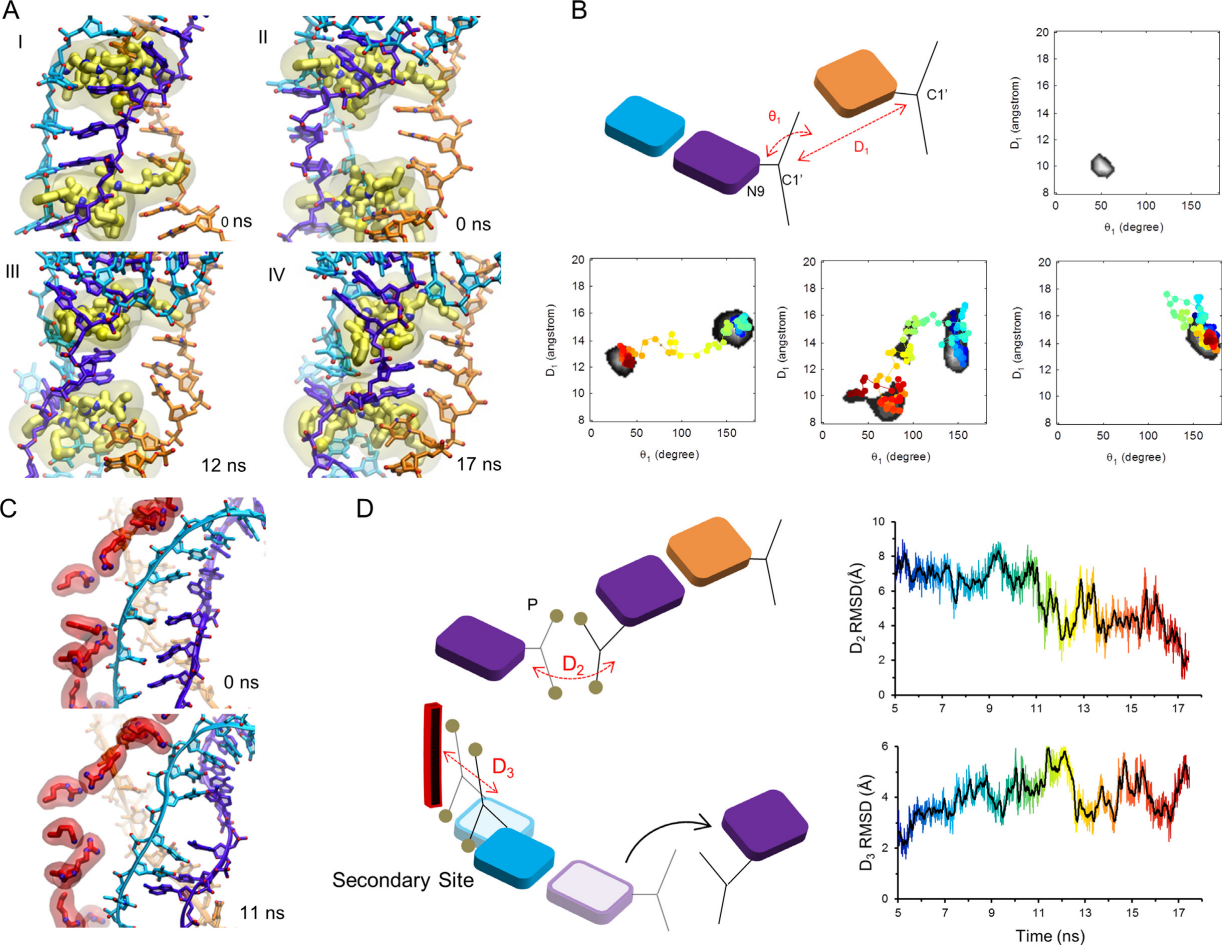
Trajectories for structures with 2 base pair triplets initially bound to site II. (**A**) I. Side view of the dsDNA. II. Rotated 90^o^ view of I. III. Structure at a later simulation time when the duplet at the 5′ end has begun to flip. IV. Flipped duplet paired with the initiating strand. The flipping of the base at the 3′ end is sterically hindered by the L2 loop. (**B**) Base pairing parameters D1 and θ_1_ are the C1′ and C1′ distance between complementary and initiating strands and C1′–C1′–N9 projection angle onto the base-flipping plane, respectively. The graphs show the histogram of D_1_ and θ_1_ for a paired dsDNA in the stable postsynaptic filament (Supplementary Figure S9). The bottom three histograms show results for a triplet bound in the site II. The left and middle one corresponds to the base-flipping process shown in (A). (**C**) Illustration of the separation of the outgoing strand from the site II residues that occurs as the bases begin to flip. Top panel illustrates the initial time and bottom panel is 11 ns later when the bases are flipped, the outgoing strand is locally separated from the site II, and in the region near the flipped duplet the complementary strand is nearly in the postsynaptic conformation. Accelerated MD was carried out after 10 ns of conventional MD simulation. (**D**) Top diagram illustrates the backbone of the complementary progress toward the position of the postsynaptic filament. Bottom diagram shows the measured distance of the outgoing strand locally separating from site II.

We decided to further explore the potential energy landscape associated with structures with two dsDNA triplets bound to site II using accelerated MD simulations (43). This technique, where the depth of potential energy wells is lessened with no a priori choices regarding variables or pathways, accelerates the passage of energy barriers to enhance the exploration. The results of this simulation are described below and in Figures 4 and 5. The trajectory after the first 10 ns can also be seen in Supplementary Movie (last scene).

Interestingly, base flipping that gave way to Watson-Crick binding in site I (shown in Figures 4 and 5) only occurred when two bases collectively flipped as a stacked duplet. Transient flipping of a unique base did not produce any binding in site I. Moreover, when base flipping resulted in Watson-Crick pairing interactions with the initiating strand, the base flipping events were locally accompanied by relocations of the dsDNA backbones (Figure 5). Relocation of the outgoing strand backbone was spatially limited but involved the transient loss of a salt bridge between the phosphate in 5′ of the flipped duplet and the corresponding R245. This salt bridge was eventually recovered after stabilization of the duplet binding in site I. Relocation of the complementary strand backbone involved large conformational changes at the duplet location, locally leading the backbone close to the position it occupies in the crystal structure of the site I-bound duplex DNA. It remained at that position until the end of the simulation (Figures 4B and 5D). The flipping of duplets occurred iteratively in the 5′ to 3′ direction, starting from the site II-bound triplet closest to the kinked junction. The second duplet only started to flip once the first duplet was stably bound (Supplementary Figure S8). Interestingly, the third base of each triplet did not flip due to steric hindrance from loop L2 (Figure 5A and Supplementary Figure S4); it remained positioned in site II where it appeared to participate in a series of stacking interactions in the dsDNA together with the two bases of the outgoing strand that lost their pairing partners. It can also be noticed that the outgoing strand partner of the 3′ base was looped out from the distorted helix in site II and firmly interacted with nearby R243. Such interactions were observed in all the triplets bound to site II and appeared to be a constant feature in the simulations, independently of the starting point. An important feature of the simulated base pairing exchange process is that the duplet that flipped towards the initiating strand remained confined in a hydrophobic environment throughout the homology testing: both the ‘top’ base at the 5′ side and the ‘bottom’ base in 3′ showed strong interactions with hydrophobic residues of the L2 loop, respectively with F203 and with M202, G200 and V201 (Figure 5A). The flipping movement can therefore be compared to sliding between two parallel air cushions, which greatly reduces the required energy when compared for example with the base flipping occurring due to thermal fluctuations. In the latter case, the flipping base not only needs to break its Watson–Crick hydrogen bonds but also loses most of its stacking interactions and gets immerged in an unfavorable aqueous environment. In the present case, the Watson-Crick bonds that needed to be broken were already greatly destabilized by the high level of stress in the site II dsDNA before the bases started to flip and the bases remained protected from water during flipping. These considerations explain why base flipping could be observed during our MD simulations only covering tens of nanoseconds, instead of ms for spontaneous flipping in isolated B-form DNA.

## DISCUSSION

### Factors supporting the starting structure

In this work, we have modeled intermediate structures in the early recognition and strand exchange processes of homologous recombination using several modeling techniques. We then evaluated starting structures using MD simulations of the evolution of the structures to determine whether they naturally lead to strand exchange processes that are compatible with homology testing. A rather complex process led us to the starting structures that we considered in the MD simulations. The unguided evolution of the starting structures with B-form dsDNA bound to lysines in the C-terminal domain that are linked to dsDNA bound to site II via kinked regions revealed both well-known structural features that were not incorporated in the model as well as new features that were unanticipated but provide insight into how the speed/stability stringency paradox can be overcome, as we will discuss in detail below.

The present work explicitly concerns the first rapid initial interactions that lead to homology testing; consequently, the speed of pairing exchange has been a major consideration when constructing the starting structure. During the construction of the starting structure, we privileged the conformations that would require as little protein movement as possible. Indeed, during the simulated base pairing exchange pathway, the protein structure almost did not vary, and the whole conformational change was concentrated on the DNA strands. Furthermore, the simulations showed that the very tense conformation of the dsDNA in site II facilitates base flipping by destabilizing its internal pairing and stacking interactions. Following its binding to site II, the dsDNA functioned like a spring whose tension increases with the number of added triplets, consistent with the observation of Danilowicz *et. al*. (17) from single molecule pulling experiments. We propose that these intermediates correspond to the quick stage of recognition and lead to the metastable 8 nt strand exchange intermediate that has been experimentally detected (11). In what follows we provide a detailed discussion of support for the major features of the structures shown in this work.

The most important support for the major features of the starting structures and their subsequent evolution is that the strand exchange occurred if duplets moved to their known postsynaptic positions, without any a priori guiding. That shifting included both base flipping and backbone relocation. Remarkably, base flipping and backbone relocation were reproduced at two levels of the filaments in successive triplets that had different structural environments, the first triplet being situated between the kinked junction in the 5′ direction and a site II-bound triplet in 3′ while the second triplet was situated between a partially flipped triplet in the 5′ direction and the dsDNA region that links the two site II bound triplets to the B-form dsDNA at the 3′ end of the filament. Evidence for the flipping of a third successive duplet situated in that dsDNA linking region in 3′ was also observed, although this duplet was too close to the filament end for any end effect to be ruled out. This reproducibility suggests the simulated process relies on general mechanical properties of the distorted double helix in the starting structure that drive evolution toward the known crystal structure of the dsDNA in the postsynaptic filament.

Further support for the starting structures is provided by the sensitivity of the evolution to features of the starting structures where the sensitivity is in good agreement with known features of the RecA system. For example, the simulations suggest that the stability of the base pairing decreased with the number of triplets bound to site II, which is consistent with base tension increasing with the number of bound triplets as a result of the differential extension between the outgoing and complementary strand backbones (17). Furthermore, B-form dsDNA ends are present in vivo, and the simulations suggest that they are required in order for the local evolution to the postsynaptic conformation. In addition, even structures with B-form tails did not show strand exchange unless there were exactly two triplets bound in site II. In that structure 8 bp are positioned to rapidly undergo strand exchange without significantly disrupting the protein structure or the structure of the dsDNA that is not bound to site II. Thus, an initial rapid test of 8 bp emerges naturally from the sensitivity of the evolution to the number of base pairs bound to site II in structures with B-form tails.

Additional support for the starting structure comes from a direct comparison of the structure itself with structural features of the RecA system that are known from experiment, but were not explicitly included in the modeling. First, the distance between the DNA strands and the helix axis agrees with the experimentally measured distance using fluorescence energy transfer by the Singleton group (5) even though attribution of the strand identities in their work differs from what we propose in the present model. Second, the model also features a sharp change in the DNA direction, consistent with observations of Rad51 strand exchange intermediates (49). Third, the spontaneous base flipping shown in the simulations is consistent with previous suggestions that base flipping is part of the recognition process (1,4). Fourth, the relocation of the complementary backbone is consistent with FRET experiments showing that backbone relocation occurs during strand exchange (5). Fifth, the flipping of a duplet that occurred while the third base from the initial triplet remained in nearly the original location is consistent with FRET data indicating that some complementary strand bases remained in the initial positions, after other complementary strand bases had already paired with the incoming strand (4,5). All of these known structural features of the dsDNA emerged naturally from unguided evolution of the starting structures.

The starting structures are also supported by comparing the roles played by protein residues during the simulations and the functional roles of the residues that are known from previous studies. For example, previous experiments suggested that the ‘gateway’ to homology recognition is the CTD surface of RecA subunits (51,52). Specifically, Shibata and colleagues showed that CTDs, and particularly exposed regions on the CTDs bearing a cluster of lysine residues, bind dsDNA (52). Among the CTD lysine residues, K286 and K302 are particularly well-conserved in bacterial RecA protein, and mutations of these residues impede strand exchange without significantly suppressing active filament formation. Mutations of less conserved lysine residues K280 and K282 also showed some suppression of homologous pairing activity (51). In addition, the intercalation of F203 into the rises in dsDNA triplets bound to site II is consistent with previous studies. In particular, proteins show some functionality when F203 is replaced by tyrosine or tryptophan (53) and conserved residue studies showed that in 8 strains F203 was replaced by the functionally similar double ring structure (54). Finally, the dsDNA binding interactions with site II residues contribute to the untwisting of the dsDNA (55). As explained above, this information on residues function could not be used directly due to the profusion of the identified residues along the whole filament and the incertitude about the sequence of events in which they are involved; however, the good agreement between the modeling and the residue studies provides additional support for the modeling, and our model proposes a plausible scenario for the time course of their involvement.

### Implications of the starting structures

As discussed below, our simulations also brought new and unexpected features that not only provide structural interpretations for many observations formerly published on the system, but also offer a solution to the speed/stability/stringency paradox as it applies to searches over a bacterial genome. The most important result that had not been anticipated before the simulations was the observation that the bases flip in duplets. Though the base pairs in both site I and site II are divided into triplets, the L2 loop sterically hinders the flipping of a complete triplet, but a duplet can easily flip. Thus, the observation that the bases do not flip in triplets does not depend on the evolution of the MD simulations; it is a basic feature of the starting structures that were obtained using several independent modeling techniques. The MD simulations merely confirmed that the duplets can indeed flip.

In addition, even without MD simulations, the starting structures suggest that during the initial stage of recognition, the dsDNA binding to the filament is dominated by interactions with dsDNA backbone phosphates. Furthermore, the B-form regions maintain the interaction between the complementary and outgoing strands even if there is no base pairing between those strands in the homology testing region. Thus, during the initial homology testing stage the nucleoprotein filament forces all three strands to remain in positions where strand exchange or reverse strand exchange can occur. Finally, base pairing in the initial strand exchange conformation can be fairly weak even for homologous pairings, while still allowing homologous pairings to remain bound long enough to progress with high probability to an irreversible strand exchange product. The influence of the B-form dsDNA ends had not been anticipated in previous works.

### Implications of the MD simulations

Other features of the model emerged only from the MD simulations. For example, the large backbone distortion that results from the flipping of a base pair duplet had not been anticipated previous to the simulations. We note that the simulations suggest that the base pairing of the flipped duplets is much less stable than the base pairing characteristic of complete triplets in the postsynaptic conformation. This difference in stability is consistent with homology testing progressing much faster in transition states containing flipped duplets than in conformations containing complete triplets in the postsynaptic conformation. The MD simulations provided a possible structural basis for the large base pairing instability that must characterize interactions during the initial rapid testing stage.

The sequential flipping of successive duplets is another feature that had not been anticipated. Importantly, it eliminates the large entropic barrier to strand exchange that would be present if 8 bases are allowed to flip individually in any order (21). Flipping in duplets may produce additional advantages since previous work has suggested that the presence of a mismatch will destabilize the pairing of neighboring bases (56). Thus, the sequential duplet flipping revealed by the simulations offers significant speed and stringency advantages over other testing strategies.

**Figure 6.**
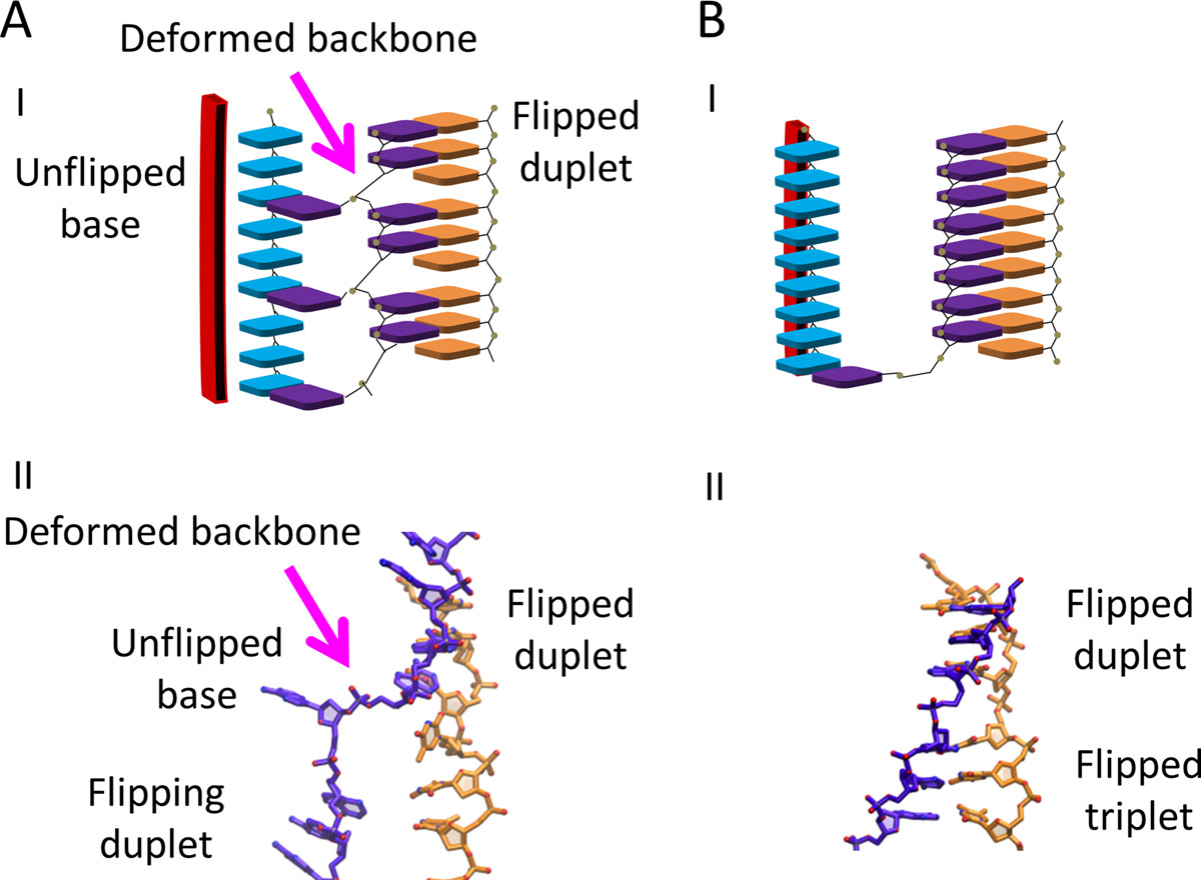
Proposed transition to the more stable configuration. The outgoing, complementary, and initiating strands are shown in cyan, purple, and orange respectively. (**A**) I. Schematic of the base pairing in a structure with three successive strand exchanged duplets. The red region indicates the position of the site II residues. (A) II. A structure showing one flipped duplet in nearly the postsynaptic conformation, followed by an unflipped duplet, followed by a duplet beginning to flip. The resulting distortion of the complementary strand backbone is visible. (**B**) I. Schematic of a conformation with 2 complete triplets in the very stable postsynaptic conformation. (B) II. Same as II. in (A), but with all of the bases in the postsynaptic conformation.

The MD simulations also provide a possible mechanism for the increase in stability that occurs once eight contiguous base pairs have undergone strand exchange. The MD simulations suggest that three successive duplets rapidly flip, leaving two unflipped bases separating the flipped duplets, as illustrated in Figure 6. If the two unflipped bases would flip in turn, then eight contiguous bp would assume the postsynaptic filament conformation and occupy the post strand exchange conformation, which would greatly reduce the mechanical stress on the complementary strand backbone, as illustrated in Figure 6. In addition, given that flipped duplets are not very stable, but structures with complete triplets in the postsynaptic conformation are highly stable (15) the flipping of the two unflipped bases would be expected to produce a great increase in the base pairing stability. None of the simulations ran long enough to observe that flipping. We anticipate that this may require some noticeable conformational change within the system, such as loop L2 displacement. Thus, results of the MD simulations are consistent with initial homology testing rapidly occurring in structures with 8 bp in a position to undergo strand exchange, where passing that homology test would lead to a transition to a much more stable strand exchange conformation.

Finally, the MD simulations show that in those structures 8 bp can undergo strand exchange while preserving the binding of B-form dsDNA at the two ends. A return to B-form dsDNA could still maintain the binding of the dsDNA to the C-terminal domains. Given that the C-terminal domains are flexible (Supplemental Figure S10), additional homology testing in a different registration could occur without changing the binding at the C-terminal domain. This would allow testing of ∼4 different registrations. Simulations also suggest that binding can be transferred from domain to domain. Such transfer of dsDNA binding between C-terminal domains might be consistent with experimental results described as ‘sliding’ (56). These two mechanisms for testing multiple registrations without free diffusion would make homology testing much more rapid than the case where the dsDNA must freely diffuse before another registration could be tested. The detailed mechanism for testing without diffusion was another unanticipated result that emerged from the model being presented here. Speculations on possible mechanisms for simultaneously testing multiple sites (57) (see also Supplementary Data) or for rejecting base pairings involving repeated sequences extending over more than 100 bp are presented in Supplementary Information.

**Figure 7.**
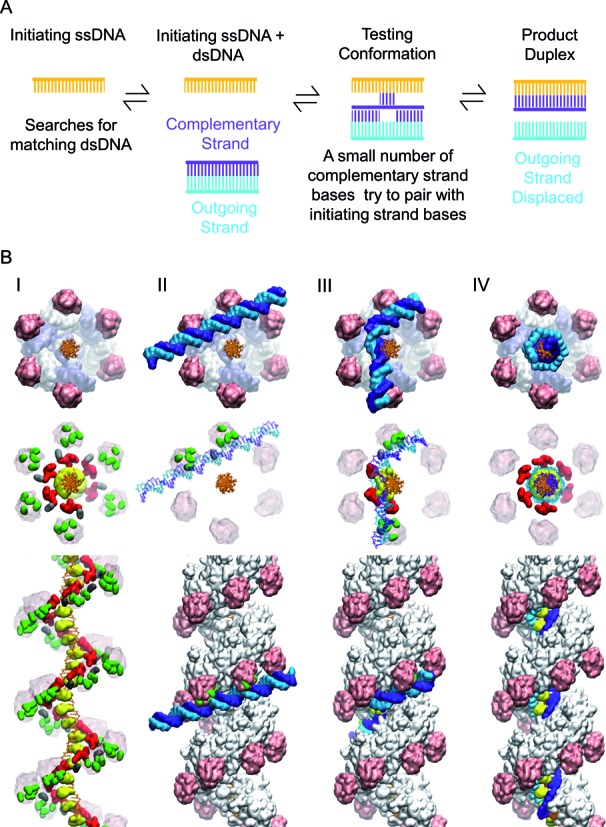
Overview of the proposed searching process. (**A**) Illustration of the known homology recognition process. The initiating, complementary, and outgoing strands are shown in orange, purple and cyan, respectively. (**B**) Overall structural transition during homology testing including the known structures (I and IV) and structures obtained using modeling and simulations (II and III). We propose that the structures correspond to the illustration shown above them in (A). The upper and central panels of (B) show end views of the filament, while the bottom panels show side views. The residues in the C-terminal domain are shown in pink except for lysine residues K280, K282, K286, and K302 which are shown in green. K232 is shown in silver. Site II residues R226, R227, R243, and K245 are shown in red, and the L2 loop (198–206) is shown in yellow.

In conclusion, the present work offers the particularity to explicitly consider the intrinsically multiscale character of the HR reaction by integrating detailed structural and functional information obtained via modeling approaches with physical models of homology search (Figure 7). This is an unusual strategy, but we propose that the high level of integration between the different aspects of the recognition puzzle is a key element for successfully studying a complex biological system that evolves on various timescales. The features of the model are in good agreement with previous experimental results. All new features obtained during the construction of the intermediate species or revealed by molecular dynamics simulations were systematically tested and/or incorporated in the theoretical model of recognition, leading to further improvements of this model. We are conscious that this body of evidence is not a direct proof that the proposed model elucidates the structural details of the mechanism of DNA sequence recognition and strand exchange. Nevertheless, we believe that owing to the intrinsic multi-scale and dynamic character of the process, our proposed model represents a formidable template to tailor new experiments that will finally establish the mechanism.

## Supplementary Material

SUPPLEMENTARY DATA
