# Supplementary material for: Integrating multi-scale data on homologous recombination into a new recognition mechanism based on simulations of the RecA-ssDNA/dsDNA structure
Source: Nucleic Acids Res. 2015 Sep 17;43(21):10251–63. doi: 10.1093/nar/gkv883 (PMC4666392; doi:10.1093/nar/gkv883)
Supplement: SUPPLEMENTARY DATA [file supp_43_21_10251__index.html]

Integrating multi-scale data on homologous recombination into a new recognition mechanism based on simulations of the RecA-ssDNA/dsDNA structure — Integrating multi-scale data on homologous recombination into a new recognition mechanism based on simulations of the RecA-ssDNA/dsDNA structure — SUPPLEMENTARY DATA 

# Integrating multi-scale data on homologous recombination into a new recognition mechanism based on simulations of the RecA-ssDNA/dsDNA structure

## SUPPLEMENTARY DATA

- SUPPLEMENTARY DATA
- SUPPLEMENTARY DATA
