# Supplementary material for: Integrating multi-scale data on homologous recombination into a new recognition mechanism based on simulations of the RecA-ssDNA/dsDNA structure
Source: Nucleic Acids Res. 2015 Sep 17;43(21):10251–63. doi: 10.1093/nar/gkv883 (PMC4666392; doi:10.1093/nar/gkv883)
Supplement: SUPPLEMENTARY DATA [file supp_gkv883_nar-01701-n-2015-File009.pdf]

Integrating multi-scale data on homologous recombination into a new recognition mechanism based on simulations of the RecA-ssDNA/dsDNA structure

Darren Yang[1], Benjamin Boyer[2], Chantal Prévost[2], Claudia Danilowicz[3], Mara Prentiss[3,*]

[1] School of Engineering and Applied Sciences, Harvard University, Cambridge, MA 02138, USA
[2] Laboratoire de Biochimie Théorique, CNRS UPR 9080, Univ Paris Diderot, Sorbonne Paris Cité, IBPC, Paris, France
[3] Department of Physics, Harvard University, Cambridge, MA 02138, USA

* To whom correspondence should be addressed. Tel: 1-617-495-4483; Fax: 1-617-495-0416; Email: prentiss@fas.harvard.edu

Supplementary Data

Parallel searching may decrease search times non-linearly

Finally, recent experimental work has indicated that the active filament probes multiple dsDNA positions in parallel (1). In such a parallel search, deep kinetic traps may barely increase searching times even if they last more than 1000x longer than the quick initial stage. Such long binding times may not greatly affect the overall searching time because the other parallel searching points may continue their rapid probing while one particular region of the active filament is stuck in a deep kinetic trap. In contrast, if the search were not parallel, the system could not test any other possible registration until the deep trap had unbound. Thus, in the presence of deep kinetic traps, a system with N parallel contact points may have a search time which is much shorter than 1/N x the searching time for a non-parallel search.

Possible strategy for rejecting pairings involving regions of accidental homology extending over 100 bp

Bacterial genomes can contain more than 10,000 repeated sequences that extend over more than 100 bp. Pairings involving such sequences could not be eliminated using the strategies considered in the main paper. Recent theoretical work (2) and the new experimental single molecule studies discussed above suggest that in the presence of ATP hydrolysis RecA-ssDNA filaments can form superstructures such as superhelices that offer the possibility for dsDNA to be aligned in registration with the ssDNA in the filament at multiple different positions. Such superhelical structures can consist of 60 RecA monomers per (or 180 ssDNA nucleotides) per superturn (2). In what follows we speculate about how RecA mediated homology recognition could exploit such superstructures to discriminate against pairings between long repeated sequences. An important feature of the proposed model is the change in the rate at which strand exchange occurs once the system makes the transition to the metastable conformation.

Once an 8-nucleotide test region of the active filament has found a homologous 8 base pair region in the dsDNA, a metastable conformation is formed (3). After the transition to the metastable conformation, strand exchange proceeds at ~ 6 bp/sec, but the strand exchange product remains fairly unstable until it extends to at least 17-20 bp. We note that *in vivo* chromosomes display significant relative motion that is absent *in vitro* (4).

We speculate that if the pairing is in registration during the ~ 2 seconds required to complete the strand exchange of ~ 20 bases, the active filament will have made in-registration contact with homologous dsDNA at multiple positions separated by 180 bp. In contrast, if the pairing involves a region of accidental homology that extends over < 180 bp, then no additional in-registration contacts will be made. If one assumes that *in vivo* the postsynaptic filament is only stable if several separate ~ 20 bp regions have undergone strand exchange, such a system would allow repeated sequence regions to be rejected even if they extended over more than 100 bp. Such a system could also greatly accelerate strand exchange since it would be starting at multiple positions.

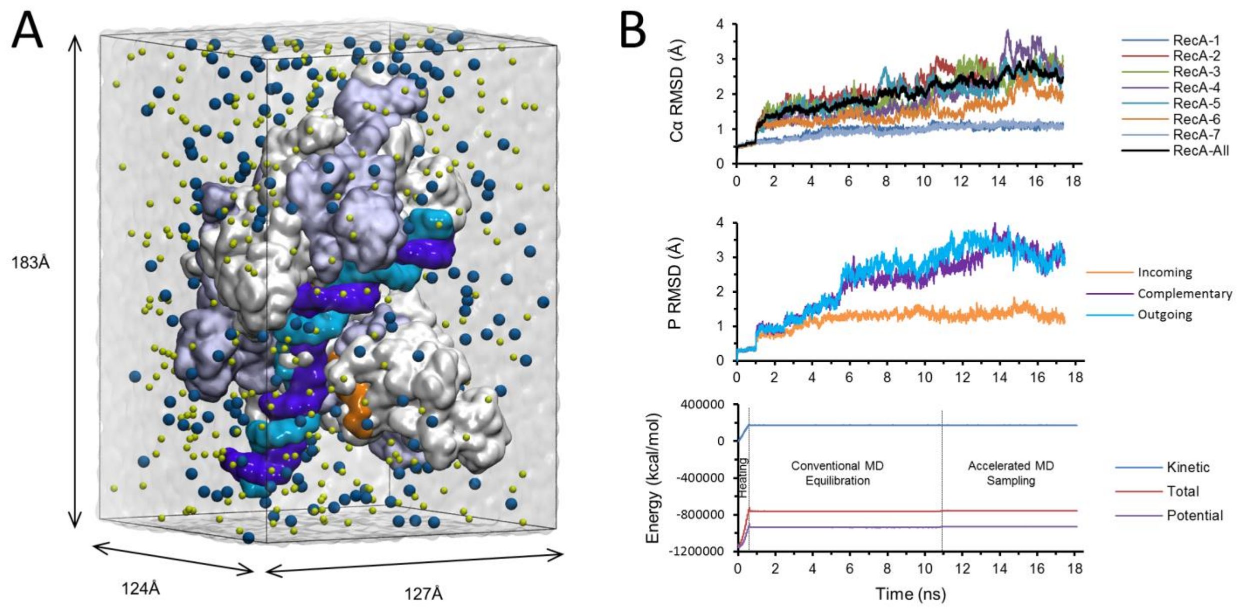

Supplementary Figure S1. Molecular dynamic simulation setup. (**A**) Schematic of a typical molecular dynamics simulation system. The solvation water is shown as the transparent box, and the sodium and chloride ions are shown as green and blue spheres, respectively. The surf molecule is the RecA-DNA complex. (**B**) RMSD and energies measured during the heating, equilibration, and production simulation.

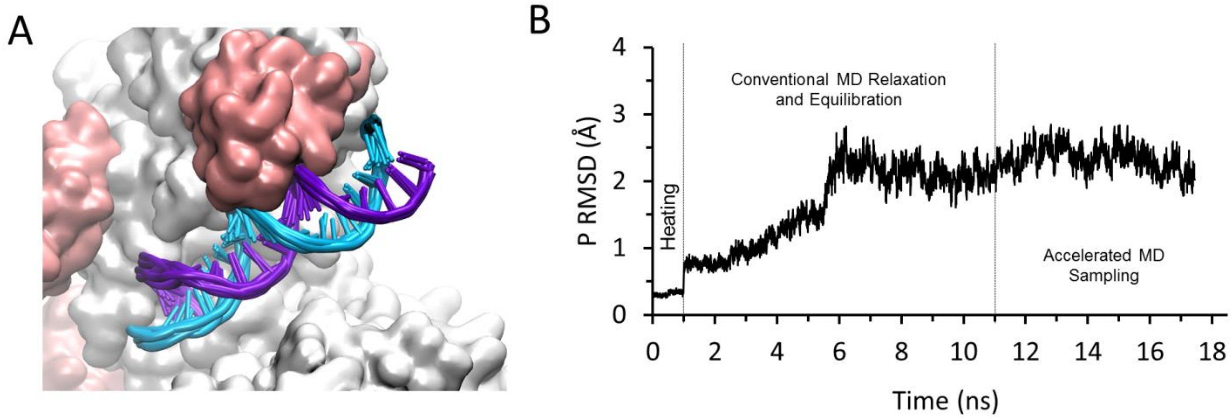

Supplementary Figure S2. Stable binding of B-form dsDNA to the surface of the C- terminal domain (CTD). (**A**) Simulation of the B-form dsDNA bound to the surface of CTD. The L2 loop, complementary, initiating, and outgoing strands are shown in yellow, purple, orange, and blue, respectively. The snapshots of the trajectories are overlapped with 3 ns simulation time gaps. (**B**) Phosphate position RMSD from the starting structure.

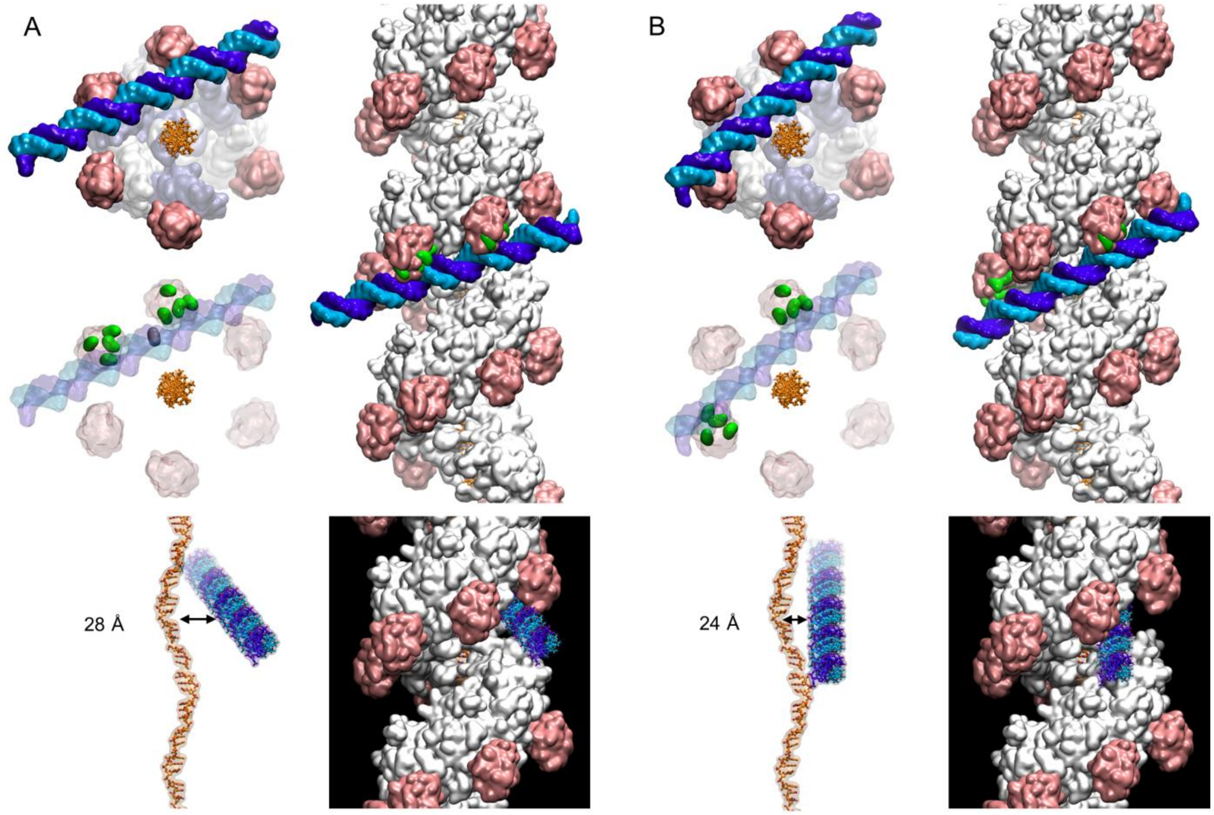

Supplementary Figure S3. Structures obtained by docking B-form dsDNA with the presynaptic filament. The structures show that the large difference in direction between the dsDNA which is nearly parallel to the open grooves in the protein filament and the ssDNA which is alinged with the central axis of the protein strongly limits the number of bases that could undergo strand exchange. The initiating, complementary, and outgoing strands are shown in orange, purple, and cyan, respectively. The residues in the C-terminal domain 270–333 are shown in pink except for lysine residues K280, K282, K286, and K302 which are shown in green. K232 is shown in silver. The remaining residues in the protein are shown in white or ice blue. (**A**) Interaction where the dsDNA is bound to neighboring CTD, where the closest backbone separation between the initiating and complementary strands is 28Å. (**B**) Interaction where the dsDNA is bound to C-terminal domains that are separated by a RecA monomer away, allowing a minimum backbone separation of 24 Å, which only applies to a single base. All other bases have larger separations. A structure with B-form dsDNA bound to C-terminal domains separated by two RecA domains is not possible because it would require a very large deformation of the stiff protein core; however, the binding is possible if the dsDNA bends, as shown in Figure 3 of the main text.

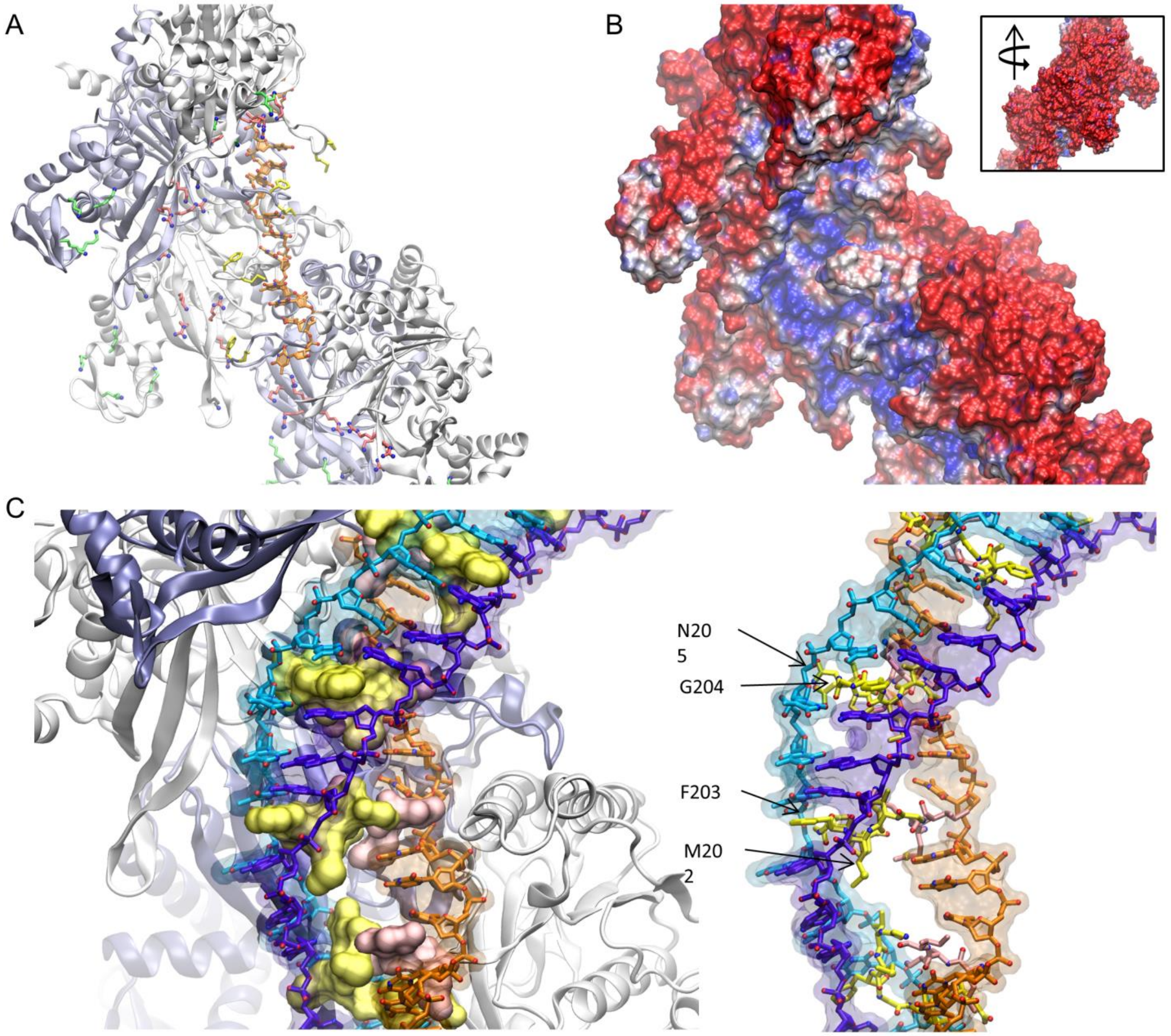

Supplementary Figure S4. Site II of the active RecA filament. (**A-B**) Residue detail and electrostatics of the secondary DNA-binding site (site II). The initiating strand is shown in orange, lysine residues K280, K282, K286, and K302 of the C-terminal Domain (CTD) are shown in green, site II residues R226, R227, R243, and K245 are shown in red, and the L2 loop residues M202 and F203 are shown in yellow. The electrostatic potential calculation was carried out by using the APBS package; the potential value is colored from red to blue with the value of (-3 to 3 in units of kT/e). (**C**) dsDNA bound to secondary site complex. The initiating, complementary, and outgoing strands are shown in orange, purple, and cyan, respectively. L2 loop residues 202-205 are shown in yellow and residues 197-199 are shown in pink. The remaining residues are shown in white and ice-blue indicating successive protein monomers. Multiple L2 loop residues 202-205 (shown in yellow) interact directly with the bound dsDNA.

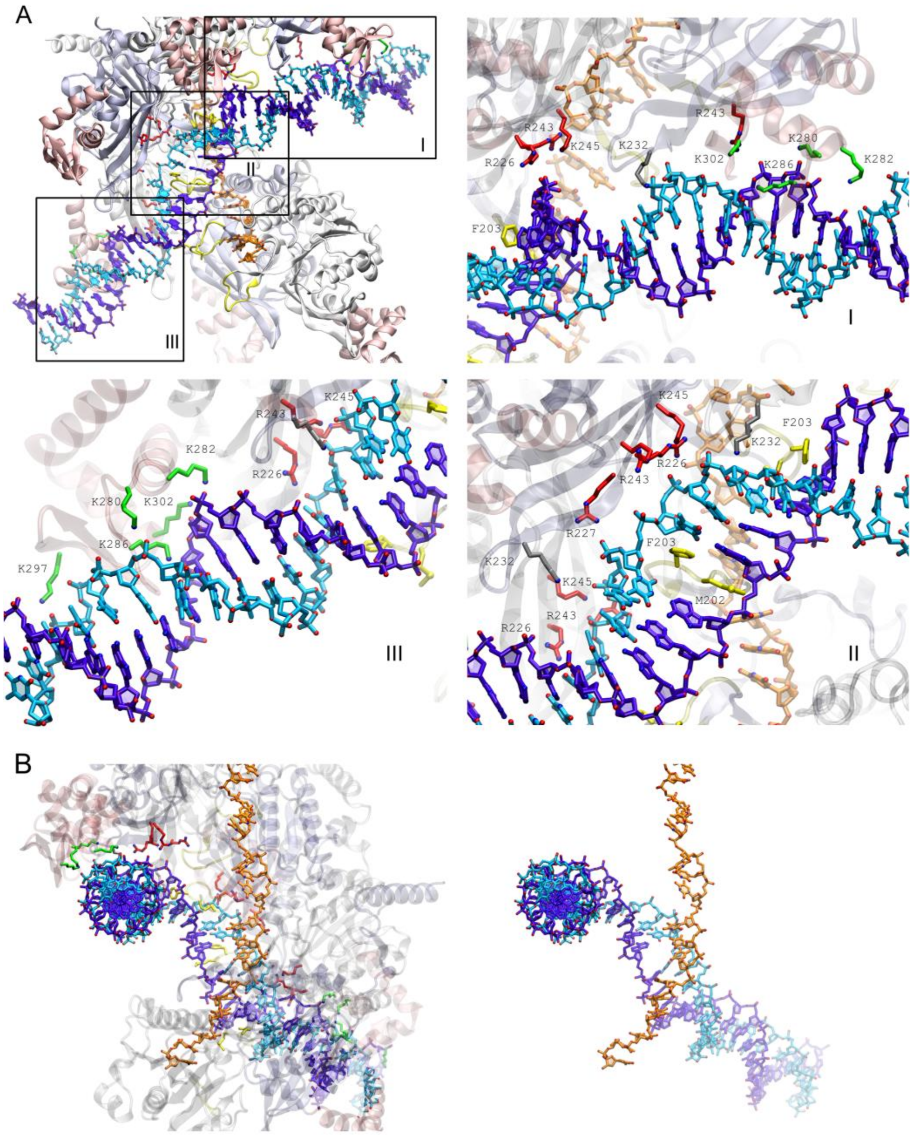

Supplementary Figure S5. Detailed view of bent dsDNA bound to active filament of RecA. The initiating, complementary, and outgoing strands are shown in orange, purple, and cyan, respectively. The residues in the C-terminal domain 270–333 are shown in pink except for lysine residues K280, K282, K286, and K302 which are shown in green. K232 is shown in silver. The secondary DNA-binding site residues R226, R227, R243, and K245 are shown in red, and the L2 loop (198-206) is shown in yellow. The remaining residues in the protein are shown in white or ice blue.The contacts with the C-terminal lysine residue are approximately the same as in B-form dsDNA as shown in Figure 3 (main text). On the 5´ side of the bend, the complementary strand has a strong interaction with R243, whereas on the 3´ side of the bend the R243 in the next monomer interacts strongly with the outgoing strand. On the 3´ side of the bend, ~ 3 bp occupy locations where the backbone spacing between the incoming and complementary strands permits base flipping to produce strand exchange.

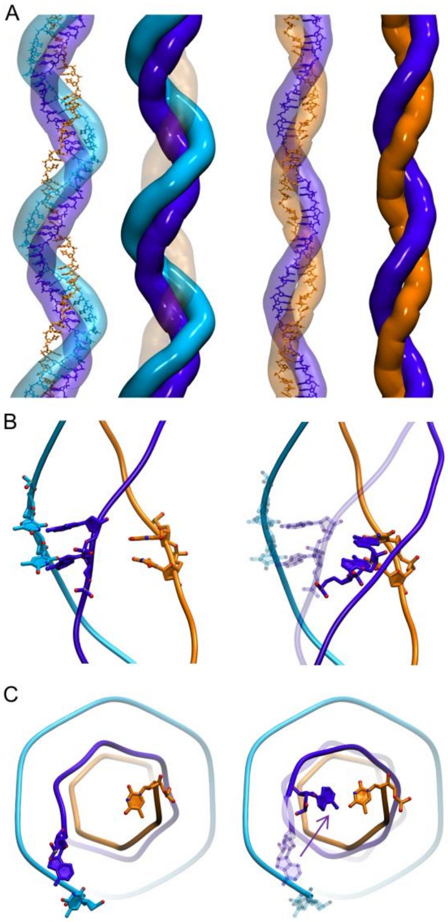

Supplementary Figure S6. Structures of modeled secondary site bound dsDNA in active filament and site I bound dsDNA in the postsynaptic filament. (**A**) The initiating, complementary, and outgoing strands are shown in orange, purple, and cyan respectively. The illustrations highlight the large extension difference between the outgoing strand in the secondary site and the initiating strand in the primary site. The first structures show the base pairing within the transparent cylinders. The second structure shows the strands as solid cylinders. These representations resemble the prediction of the previous experimental work by Egelman *et. al.* (5). Egelman proposed 3 backbone positions within the RecA filament corresponding to backbones with radii of 15Å, 10Å, and 6Å, and suggested that though all three proposed structures had a 5.1 Å rise/bp along the helical axis, the 15 Å radius structure (corresponds to the blue identical strand) had a 7.2 Å separation between phosphates, which corresponds to the maximum possible backbone extension. In contrast, the 10-Å and 6-Å radius structures were projected to have separations of only 6.1 and 5.5 Å, respectively. (**B**) Side view of corresponding base pair duplet is shown for the two structures. In the postsynaptic filament on the right, the complementary base position prior to base-flipping is shown by a transparent rendering. (**C**) Bottom view from the 3´ to 5´ end. The illustrations also show that the dynamics evolution toward postsynaptic structure is not simply a matter of base flipping: substantial complementary strand backbone relocation is also required.

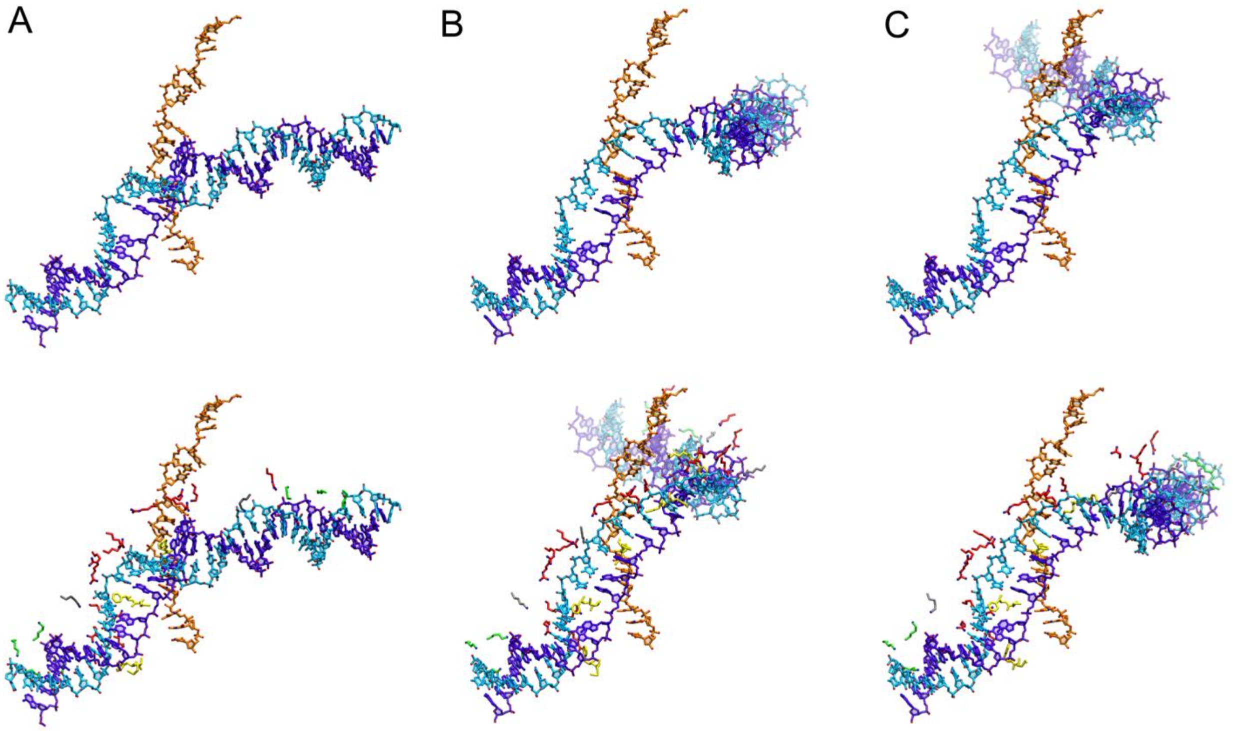

Supplementary Figure S7. Structures consisting of B-form tails attached to dsDNA untwisted and extended by binding to site II. The number of bp bound to site II increases from left to right (**A)** to (**C**). The colors and the first figure are the same as those used in Figure 3 and Supplementary Figure S5.

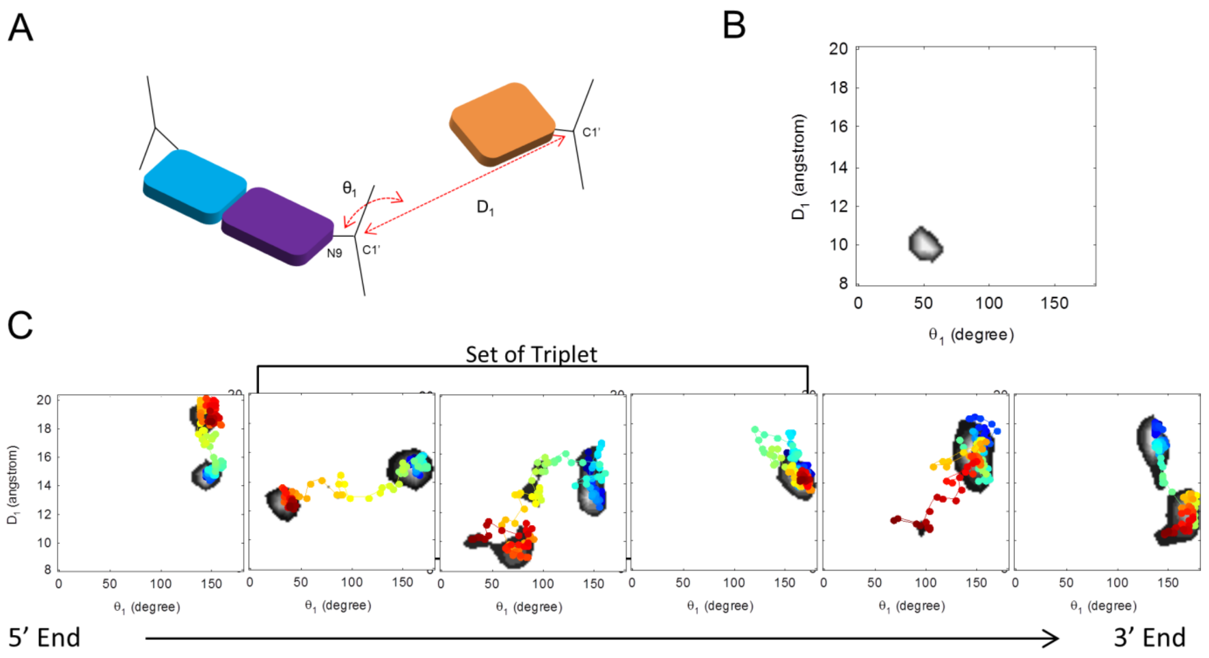

Supplementary Figure S8. Evolution of a complementary strand duplet flipping from pairing with the identical strand to pairing with the initiating strand. (**A**) Base paring parameters D_1 and θ_1 are the C1´ and C1´ distance between complementary and initiating strands and the C1´-C1´-N9 projection angle onto the base-flipping plane, respectively. (**B**) Histogram of D_1 and θ_1 of a paired dsDNA in the stable postsynaptic filament obtained from MD simulation (see Supplementary Figure 9). (**C**) Histograms and trajectories (blue to red) illustrating base-flipping parameter of six bases from three triplets bound in the secondary site. The second and third diagrams correspond to the flipped duplex as shown in the main text. The last two diagrams illustrate the early stage of flipping.

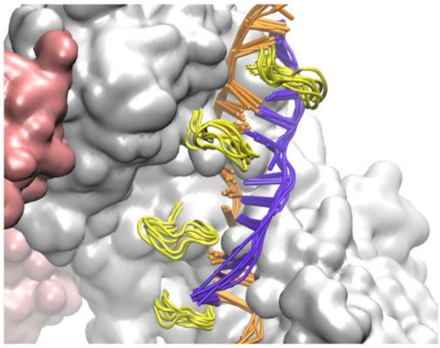

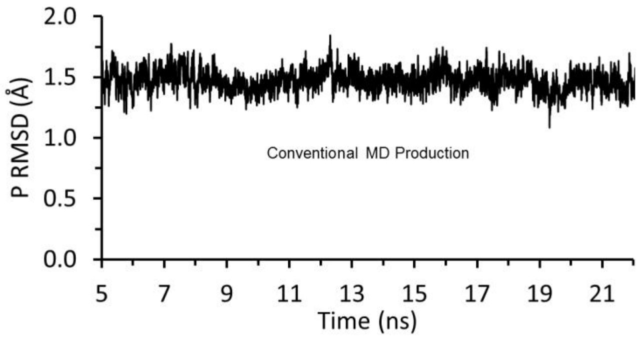

Supplementary Figure S9. **Stable binding of dsDNA in the primary site.** Simulation of the postsynaptic filament with the post strand-exchange dsDNA bound to the primary site (site **I**). The phosphate position RMSD from the initial starting structure during production simulation is shown on the right. The average RMSD of the DNA phosphate atom is ~1.5 Å.

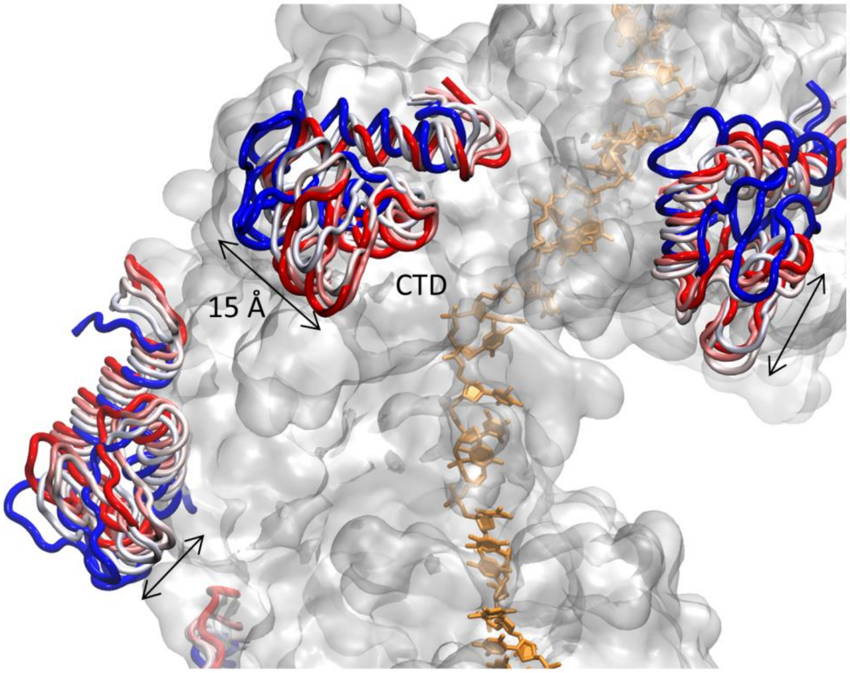

Supplementary Figure S10. Illustration of the flexibility and range of fluctuation of the CTD. Trajectories of the CTD during the 15 ns production simulation are colored blue to red with 3 ns gap. The backbone of CTD can have over 15-Å range of motion while maintaining its internal structure.

REFERENCES

1. Forget, A.L. and Kowalczykowski, S.C. (2012) Single-molecule imaging of DNA pairing by RecA reveals a three-dimensional homology search. *Nature*, **482**, 423-U178.
2. Boyer, B., Ezelin, J., Poulain, P., Saladin, A., Zacharias, M., Robert, C.H. and Prévost, C. (2015) An Integrative Approach to the Study of Filamentous Oligomeric Assemblies, with Application to RecA. *PLoS ONE*, **10**, e0116414.
3. Hsieh, P., Camerini-Otero, C.S. and Camerini-Otero, R.D. (1992) The synapsis event in the homologous pairing of DNAs: RecA recognizes and pairs less than one helical repeat of DNA. *Proc. Natl Acad. Sci. USA* **89**, 6492-6496.
4. Fisher, J.K., Bourniquel, A., Witz, G., Weiner, B., Prentiss, M. and Kleckner, N. (2013) Four-Dimensional Imaging of E. coli Nucleoid Organization and Dynamics in Living Cells. *Cell*, **153**, 882-895.
5. Egelman, E. and Yu, X. (1989) The location of DNA in RecA-DNA helical filaments. *Science*, **245**, 404-407.
